# Muscle strength, EEG biomarkers, and working memory as interacting predictors of cognitive function in cognitively impaired older adults

**DOI:** 10.3389/fnagi.2025.1641209

**Published:** 2025-09-04

**Authors:** Yagang Song, Shuqi Jia, Xing Wang, Aiwei Wang, Shufan Li, Feng Ding, Tao Ma, Xueping Wu

**Affiliations:** ^1^Department of Physical Education Teaching, Shanghai Sanda University, Shanghai, China; ^2^School of Physical Education, Shanghai University of Sport, Shanghai, China; ^3^College of Physical Education, Yangzhou University, Yangzhou, China; ^4^Faculty of Sports Science Ningbo University, Ningbo, China

**Keywords:** cognitive impairment, EEG characteristics, muscle strength, working memory, cognitive function

## Abstract

**Background:**

Cognitive decline in older adults is a pressing public health concern, with emerging evidence suggesting that both muscle strength and neural function may influence cognitive outcomes. However, the integrative mechanisms linking these domains remain insufficiently understood.

**Objective:**

This study aimed to explore whether resting-state EEG characteristics and working memory mediate the relationship between muscle strength and global cognitive function in older adults with cognitive impairment. Methods: A cross-sectional study was conducted among 137 older adults (mean age = 72.65 ± 7.75) with cognitive impairment. Muscle strength was assessed using grip strength and 30 s chair stand tests. Resting-state EEG power across six frequency bands was recorded from 16 electrodes. Working memory was evaluated using a two-back task, and cognitive function was assessed via the MoCA. Mediation analyses were performed using the PROCESS macro (Model 4), controlling for age, sex, education, and BMI.

**Results:**

Grip strength showed significant direct effects on cognitive function (*β* = 0.399, *p* < 0.001), with partial mediation by both working memory (β = 0.070, *p* < 0.05) and resting-state EEG (*β* = 0.150, *p* < 0.01). In contrast, lower limb strength was mediated only by working memory (β = 0.078, *p* < 0.05), while EEG-based mediation was not significant. The overall model explained 50.7% of the variance in cognitive outcomes.

**Conclusion:**

This study highlights the distinct mediating roles of working memory and EEG features in the muscle strength–cognition relationship. Grip strength, as a potential biomarker, may reflect central nervous system integrity and serve as a target for cognitive health interventions in aging populations.

## 1 Introduction

According to projections by the World Health Organization (WHO), the global population aged 60 years and above will reach 2.1 billion by 2050, accounting for 22% of the total population (Bajwa et al., 2019). Population aging is closely associated with increased prevalence of age-related neurodegenerative diseases (Vassilaki et al., 2025). Identifying modifiable risk factors and developing targeted interventions to mitigate cognitive decline in older adults has therefore become an urgent public health priority (He et al., 2023). Cognitive impairment in aging individuals typically manifests as deficits in memory, attention, executive function, language, and visuospatial skills (Xin et al., 2025). As cognitive capacity declines, so does autonomy—placing growing demands on caregivers and generating substantial burdens on healthcare systems and families (Connors et al., 2019).

Among the key predictors of cognitive health, working memory plays a central role, as it is particularly sensitive to age-related neural deterioration. Cognitive decline is often rooted in structural and functional degeneration of the prefrontal, temporal, and parietal cortices (Kamio et al., 2025), with working memory impairment being a common early symptom (D’esposito and Postle, 2015). From a neurobiological perspective, age-related disruptions in cortical connectivity are thought to drive this deterioration (Wang et al., 2011). Meanwhile, muscle strength—including both upper- and lower-limb performance—has emerged as a potentially modifiable factor linked to working memory and general cognition (Cai et al., 2023; Veronese et al., 2016; Chou et al., 2019). Longitudinal studies have demonstrated that individuals with reduced muscular strength in mid-to-late life are at elevated risk for cognitive impairment and dementia.

Muscle strength declines progressively with age, beginning as early as the third decade of life. By age 40, strength decreases by approximately 16%, with reductions reaching over 40% after the age of 60 (Keller and Engelhardt, 2013). Similarly, working memory peaks around age 30 and undergoes accelerated decline after 60 (Wang et al., 2011). This temporal overlap suggests a possible synchrony between neuromuscular and cognitive aging trajectories (Liao et al., 2024).

Resting-state electroencephalography (EEG)—a non-invasive technique with excellent temporal resolution—has emerged as a valuable tool for characterizing neural mechanisms of age-related cognitive decline (Puri et al., 2025). EEG captures spontaneous neural oscillations under task-free conditions, reflecting the brain’s intrinsic capacity for information processing (Si et al., 2019; Li et al., 2015). Age-associated EEG alterations include global slowing of peak frequencies, reductions in alpha-band power (8–12 Hz), and increases in delta (1–4 Hz) and theta (4–8 Hz) power across cortical regions (Klimesch, 2012; Dujardin et al., 1995; Klass and Brenner, 1995). These spectral changes are closely linked to declines in attention, memory, and executive functioning.

Taken together, the literature supports a multifactorial model of cognitive aging—one that integrates physical frailty, neural degradation, and disrupted cognitive resource allocation. Specifically, emerging evidence suggests that resting-state EEG biomarkers (as proxies for neural efficiency), muscle strength (as an index of physiological reserve), and working memory (as a core cognitive domain) may interactively influence cognitive outcomes in older adults. This framework prompts important mechanistic questions: What are the pathways by which muscular strength affects cognition? Do EEG features and working memory mediate these effects? Are the pathways consistent across different limb strengths (e.g., grip vs. lower-body performance)?

To address these knowledge gaps, the present study aimed to clarify the neurocognitive pathways linking muscle strength and cognitive function in older adults with cognitive impairment. Specifically, we posed the following research questions:1. Do working memory and resting-state EEG characteristics mediate the association between muscle strength and global cognitive performance? 2. Which specific EEG features (e.g., frontal theta power, parietal alpha frequency) are most predictive of cognitive function? 3. Do upper-limb (e.g., grip strength) and lower-limb (e.g., chair stand performance) strength indicators exert differential effects through distinct neural and cognitive pathways? Based on these questions, we proposed three hypotheses: H1: Muscle strength, working memory, and resting-state EEG parameters form an integrated predictive model for cognitive function. H2: Muscle strength is significantly correlated with both resting-state EEG features and working memory, which in turn are associated with cognitive performance. H3: Working memory and EEG spectral power jointly mediate the relationship between muscle strength and cognition.

To test these hypotheses, we conducted a cross-sectional analysis among older adults with cognitive impairment, incorporating standardized assessments of muscle strength (upper and lower limbs), working memory, and resting-state EEG. Path analysis was used to identify direct and indirect effects among these variables. Our goal is to clarify the neurophysiological mechanisms by which muscular fitness influences cognitive function, thereby informing strategies for early detection and intervention in age-related cognitive decline.

## 2 Research objects and methods

### 2.1 Sample size and power analysis

A priori power analysis was conducted using G*Power 3.1 to determine the minimum sample size required for multiple regression with three main predictors (muscle strength, EEG features, and working memory). Assuming a medium effect size (f^2^ = 0.15), an alpha level of 0.05, and desired statistical power of 0.80, the required sample size was 89 participants. The final analytic sample (*N* = 137) exceeds this threshold, ensuring adequate statistical power for detecting medium-sized effects in the mediation models.

### 2.2 Recruitment of subjects

Using convenience sampling method from March to May 2024, recruitment posters and leaflets were distributed in Chenfu Jiayuan and Qiangwei Jiuli communities of Songjiang District, and Luyan community of Jinshan District in Shanghai. A total of 145 eligible older adults were recruited based on voluntary participation. During testing, 1 participant was excluded due to exercise contraindications, 2 due to poor hearing/vision, and 5 failed to complete EEG acquisition, resulting in 137 final participants. This study complied with the latest version of the Helsinki Declaration and was approved by Shanghai University of Sport Ethics Committee (102772020RT060). This study was reported in accordance with the STROBE statement for cross-sectional studies. All participants voluntarily participated in the experiment and signed informed consent forms.

#### 2.2.1 Inclusion criteria

① Aged 60 years or older; ② Right-handedness; ③ Good physical condition; ④ Absence of severe cardiovascular diseases; ⑤ Normal vision and hearing; ⑥ Normal mental status with verbal communication ability and willingness to cooperate with investigation; ⑦ Willingness to sign informed consent. All participants were included in the analysis based on cognitive screening (MoCA score <26).

#### 2.2.2 Exclusion criteria

① Severe cardiovascular diseases or major organic diseases; ② Severe musculoskeletal disorders preventing prolonged standing; ③ Contraindications to physical exercise; ④ Long-term or recent use of medications affecting physical activity, psychotropic drugs, cholinergic inhibitors, or related substances.

### 2.3 Test process

**Figure 1:**
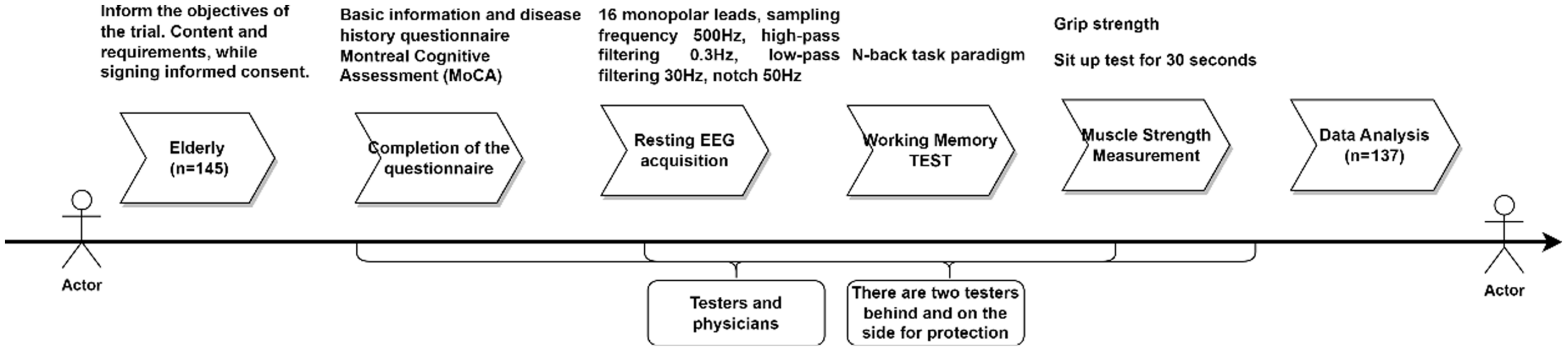
Test flow chart.

All tests were conducted between 13:30 and 16:30. Participants were required to visit the laboratory twice. During the first visit, experimental procedures were explained, and participants completed a basic information form and the Montreal Cognitive Assessment (MoCA) scale. The second visit involved EEG signal acquisition and working memory testing. Additional assessments included height, weight, handgrip strength tests, and a 30 s chair stand test. The testing procedure is shown in Figure 1. Participants were instructed to refrain from vigorous exercise and avoid consuming caffeinated or alcoholic beverages 24 h prior to testing. All participants voluntarily participated in the experiment, signed informed consent forms, and the study adhered to the latest version of the Helsinki Declaration.

#### 2.3.1 Test tool

##### 2.3.1.1 Self-designed basic information questionnaire

Collected participants’ demographic data including name, age, gender, BMI, marital status, educational level, occupation, place of residence, presence/absence of exercise contraindications, smoking and alcohol consumption history, decline in hearing or vision, social activities, medical history, and medication usage.

##### 2.3.1.2 Montreal cognitive assessment (MoCA)

Global cognitive function was assessed using the Beijing version of the Montreal Cognitive Assessment (MoCA), a widely validated screening tool for mild cognitive impairment (MCI). The MoCA evaluates eight cognitive domains including visuospatial/executive function, naming, memory, attention, language fluency, abstract reasoning, delayed recall, and orientation, with a maximum score of 30 (higher scores indicate better cognition). The cutoff score of <26 was used to identify cognitive impairment. To control for educational level, 1 or 2 points were added as recommended. The MoCA was selected over other tools (e.g., MMSE) due to its higher sensitivity in detecting early-stage MCI, especially in community-dwelling older adults. The Beijing version has been validated in Chinese populations, with a reported test–retest reliability of 0.857 (Yang et al., 2018).

##### 2.3.1.3 Muscle strength test

This study assessed muscle strength using handgrip strength and the 30 s chair stand test. Handgrip strength reflects whole-body strength and physical function with high practicality and sensitivity. Participants stood upright with arms extended by their sides, gripping a dynamometer with maximum force for 3–5 s. Each hand was tested three times (with 30 s rests between trials), and the mean value was calculated. The 30 s chair stand test demonstrates strong validity and reliability for evaluating lower limb muscle strength in older adults, and a simple assessment model using this test as reference values has been preliminarily established. Participants stood in front of a 43 cm-high chair with arms crossed over the chest. Upon starting the timer, they repeatedly stood up and sat down as quickly as possible within 30 s. Strict protocols were enforced: maintaining upright posture without touching the backrest, and requiring full knee extension during standing.

##### 2.3.1.4 Working memory test

The N-back task paradigm, a classic psychological experiment, was used to assess working memory. Stimuli (digits) were programmed using E-Prime 2.0 software. Participants responded to stimuli via keyboard according to task rules, with reaction time and accuracy recorded. Three cognitive load levels (0-back, 1-back, 2-back) were implemented. 0-back: Compare current digit with “0” – press “1” for match, “2” for mismatch. 1-back (starting from 2nd digit): Press “1” if identical to previous digit, else “2.” 2-back (starting from 3rd digit): Press “1” if matching the digit two steps back, else “2.” Instructions and practice trials preceded formal testing, with each load level repeated 5 times. Each trial began with a 500 ms fixation cross “+,” followed by random presentation of 10 digits (0–9) for 500 ms each, then a 2000 ms blank screen. Unresponded trials automatically advanced. A “Rest 30s” prompt appeared after each task block. Total testing duration was approximately 15 min.

##### 2.3.1.5 EEG signal acquisition

Resting-state EEG was recorded using a 16-channel amplifier-equipped system (NCERP-190012, Shanghai Nuocheng Electric Co., Ltd.), with electrodes placed according to the international 10–20 system at Fp1, Fp2, F3, F4, F7, F8, C3, C4, P3, P4, O1, O2, T3, T4, T5, and T6. The ground electrode was placed on GND, and signals were re-referenced offline to linked mastoids (A1, A2). Impedances were maintained below 5 kΩ. Participants were tested in a soundproof, dimly lit, and ventilated room free of sensory-stimulating devices (e.g., mobile phones). After an adaptation period, participants were seated comfortably with hands resting, eyes closed, and instructed to remain awake, relaxed, and motionless during the 5 min eyes-closed (EC) resting-state recording. EEG signals were recorded at a sampling rate of 500 Hz and filtered in real-time using a 0.3 Hz high-pass, 30 Hz low-pass, and 50 Hz notch filter. Raw EEG data were further preprocessed in MATLAB using the EEGLAB toolbox. Preprocessing steps included: Offline re-referencing to bilateral mastoids (A1, A2). Band-pass filtering between 0.3–30 Hz and a 50 Hz notch filter to suppress powerline noise. Visual inspection for gross artifacts. Independent Component Analysis (ICA) to identify and remove components related to eye movements (EOG) and muscle activity. No regression-based removal of physiological signals (e.g., ECG, EMG) was performed, and this limitation is acknowledged in the Discussion section. EEG spectral power was calculated using Fast Fourier Transform (FFT) across the following frequency bands: delta (1–4 Hz), theta (4–8 Hz), alpha-1 (8–10.5 Hz), alpha-2 (10.5–13 Hz), beta-1 (13–20 Hz), and beta-2 (20–30 Hz), using left-closed-right-open interval definitions. Frequency boundary values were assigned to the higher band. The entire 5 min EC segment was retained without epoching. Regions of interest included frontal (Fp1, F7) and central (C4) electrodes, consistent with prior studies on cognitive aging and EEG biomarkers.

### 2.4 Statistical analysis

Pearson correlation and stepwise regression analyses were used to examine the associations between resting-state EEG features, muscle strength, working memory, and cognitive function in older adults. Based on these associations, path models were constructed with muscle strength as the independent variable and cognitive function as the outcome, while controlling for age, BMI, and years of education. Two separate mediation models were specified, treating EEG spectral indices and working memory metrics as mediator variables, respectively. The PROCESS macro (v4.1) Model 4 was used to estimate direct and indirect effects with 5,000 bootstrap resamples to calculate 95% confidence intervals (CIs). All variables were standardized (z-scores) prior to modeling. Statistical significance was assessed using two-tailed tests with *α* = 0.05, where *p*-values < 0.05, < 0.01, and < 0.001 indicated increasing levels of significance. To quantify the strength of the mediation, the proportion mediated was calculated as follows:


$$\text{Mediation proportion}=\frac{\mathrm{ab}}{ab+c\prime }$$

Where *a* represents the effect of the independent variable on the mediator, *b* the effect of the mediator on the dependent variable, and *c′* the direct effect of the independent variable on the dependent variable after adjusting for the mediator. The ratio was multiplied by 100 to express the mediated effect as a percentage of the total effect. In addition, hierarchical multiple regression analysis was conducted to evaluate the incremental predictive contribution of muscle strength, working memory, and EEG features to cognitive performance. Predictors were entered in sequential blocks, beginning with demographic covariates, followed by physical, cognitive, and neural variables. Multicollinearity among predictors was assessed using the Variance Inflation Factor (VIF). A VIF close to 1 suggests no multicollinearity; values between 1 and 5 were considered acceptable; values ≥5 indicated moderate multicollinearity; and VIF ≥ 10 signaled severe multicollinearity, warranting further model diagnostics. The independence of residuals was evaluated using the Durbin–Watson statistic (DW), with acceptable values ranging from 1.5 to 2.5, indicating no substantial autocorrelation.

## 3 Results

### 3.1 Basic information of the subjects

The study enrolled 137 participants (age: 72.65 ± 7.754 years; BMI: 24.044 ± 3.258 kg/m^2^), with 56.20% male and 43.80% female. Comparative analysis of demographic characteristics between mild and moderate–severe cognitive impairment groups (Table 1) revealed statistically significant differences in age and education years (all *p* < 0.05), while other variables showed no significant group differences (all *p* > 0.05). These findings suggest that advanced age is associated with greater cognitive impairment severity in older adults, whereas longer educational attainment correlates with reduced impairment levels.

**Table 1:** Group differences in demographic variables between older adults with mild and moderate-to-severe cognitive impairment.

Variable	Total (*n* = 137)	Mild CI (*n* = 59)	Moderate–severe CI (*n* = 78)	Group comparison
Age (years)	72.65 ± 7.75	69.95 ± 6.80	74.69 ± 7.85	t = −3.71, *p* < 0.001
Education (years)	5.47 ± 3.49	6.66 ± 2.90	4.58 ± 3.64	t = 3.73, *p* < 0.001
BMI (kg/m^2^)	24.04 ± 3.26	24.47 ± 2.94	23.72 ± 3.46	t = 1.35, *p* = 0.180
Sex, *n* (%)				χ^2^ = 0.98, *p* = 0.323
Male	77 (56.2%)	36 (61.0%)	41 (52.6%)	
Female	60 (43.8%)	23 (39.0%)	37 (47.4%)	
Residence, *n* (%)				χ^2^ = 0.41, *p* = 0.523
Rural	108 (78.8%)	45 (76.3%)	63 (80.8%)	
Urban	29 (21.2%)	14 (23.7%)	15 (19.2%)	
Hearing loss, *n* (%)				χ^2^ = 2.33, *p* = 0.127
Yes	66 (48.2%)	24 (40.7%)	42 (53.8%)	
No	71 (51.8%)	35 (59.3%)	36 (46.2%)	
Vision loss, *n* (%)				χ^2^ = 0.41, *p* = 0.523
Yes	90 (65.7%)	37 (62.7%)	53 (68.0%)	
No	47 (34.3%)	22 (37.3%)	25 (32.0%)	
Smoking, *n* (%)				χ^2^ = 1.09, *p* = 0.297
Yes	49 (35.8%)	24 (40.7%)	25 (32.1%)	
No	88 (64.2%)	35 (59.3%)	53 (67.9%)	
Alcohol use, *n* (%)				χ^2^ = 2.56, *p* = 0.109
Yes	50 (36.5%)	26 (44.1%)	24 (30.8%)	
No	87 (63.5%)	33 (55.9%)	54 (69.2%)	
Falls in past year, *n* (%)				χ^2^ = 1.68, *p* = 0.195
Yes	38 (27.7%)	13 (22.0%)	25 (32.1%)	
No	99 (72.3%)	46 (78.0%)	53 (67.9%)	
Chronic diseases, *n* (%)				χ^2^ = 0.78, *p* = 0.377
Yes	96 (70.1%)	39 (66.1%)	57 (73.1%)	
No	41 (29.9%)	20 (33.9%)	21 (26.9%)	

Group comparisons were conducted using independent samples t-tests for continuous variables and chi-square (χ^2^) tests for categorical variables. CI = Cognitive Impairment; BMI = Body Mass Index. *p* < 0.05, *p* < 0.01 indicate statistical significance.

### 3.2 Model construction of resting electroencephalogram characteristics, muscle strength, working memory and cognitive function

Hierarchical stepwise regression analysis (Table 2) demonstrated a model fit of R^2^ = 0.507 (*F*(1,127) = 18.564, *p* < 0.001). In the final step of the hierarchical regression analysis, the combined predictors—muscle strength, resting-state EEG features, and working memory—explained 50.7% of the total variance in MoCA scores (R^2^ = 0.507), indicating strong overall model fit. Variance inflation factors (VIF) ranged between 1 and 5, and the Durbin-Watson statistic (DW = 1.974) fell within 1.5–2.5, both indicating acceptable multicollinearity and independence of residuals. Bootstrap validation results are presented in Table 3. The findings reveal multivariate relationships among resting-state EEG features, muscle strength, working memory, and cognitive function. These combined characteristics serve as significant predictors, providing empirical support for developing preventive strategies and predictive models of cognitive decline in older adults.

**Table 2:** Multivariate analysis of resting electroencephalogram characteristics, muscle strength, working memory and cognitive function.

Model	Standardized beta	Std. Error	t	*p*	Collinearity Statistics		Model Fit
					Tolerance	VIF	
(Constant)		2.956	0.332	0.741 (−4.869, 6.830)			R^2^ = 0.507; *F*(1,127) = 18.564, *p* < 0.001; DW = 1.974
Fp1θ	−0.24	0.109	−2.814	0.006 (−0.521, −0.091)	0.532	1.879	
F7α2	0.183	0.031	2.226	0.028 (0.008, 0.131)	0.573	1.745	
Fp1δ	0.469	0.065	4.146	<0.001 (0.140, 0.396)	0.303	3.297	
C4δ	−0.512	0.166	−4.577	<0.001 (−1.091, −0.432)	0.31	3.224	
T4α2	0.123	0.128	1.724	0.087 (−0.033, 0.475)	0.759	1.317	
O1δ	0.207	0.132	2.094	0.038 (0.015, 0.539)	0.399	2.509	
Max grip strength	0.256	0.047	3.662	<0.001 (0.078, 0.263)	0.796	1.257	
30s chair stand	0.127	0.09	1.898	0.060 (−0.007, 0.348)	0.862	1.161	
2-back task accuracy	0.284	3.882	4.309	<0.001 (9.043, 24.405)	0.895	1.117	

**Table 3:** Bootstrap test results.

Bootstrap coefficients					95% CI*	
Model	Unstandardized	Bias	Standard Error	p*	Lower	Upper
(Intercept)	0.004	0.003	0.063	0.957	−0.131	0.117
Max grip strength	0.253	−0.004	0.078	0.002	0.089	0.398
30s chair stand	0.126	−0.002	0.065	0.045	0.003	0.26
2-back task Accuracy	0.288	0.003	0.075	<0.001	0.118	0.42
Fp1δ	0.46	−0.015	0.133	0.004	0.207	0.737
Fp1θ	−0.256	−0.018	0.092	0.009	−0.412	−0.053
F7α2	0.184	0.003	0.073	0.007	0.048	0.329
T4α2	0.122	−38.8	0.065	0.054	−0.004	0.254
C4δ	−0.504	0.039	0.157	0.009	−0.74	−0.15
O1δ	0.203	−0.006	0.113	0.096	−0.057	0.405

Bootstrapping based on 5,000 replicates. Coefficient estimate is based on the median of the bootstrap distribution. * Bias corrected accelerated.

### 3.3 The realization path of cognitive function improvement in the elderly with cognitive impairment

#### 3.3.1 The relationship between resting electroencephalogram characteristics, muscle strength, working memory and cognitive function in the elderly with cognitive impairment

Correlation analyses across variables in the study cohort (Table 4) revealed no significant correlation between working memory and resting-state EEG features, while other primary variables demonstrated varying degrees of association (absolute r-values ranging from 0.201 to 0.726). The results confirm that resting-state EEG features, muscle strength, working memory, and cognitive function exhibit interconnected relationships.

**Table 4:** Matrix of correlation coefficients between resting EEG characteristics, muscle strength, working memory, and cognitive function.

Variable	1	2	3	4	5	6	7	8	9	10
1. MoCA	—									
2. Max grip strength	0.469^***^	—								
3. 30s Chair Stand	0.294^***^	0.239^**^	—							
4. 2-back task accuracy	0.432^***^	0.197^*^	0.202^*^	—						
5. Fp1δ	−0.062	−0.204^*^	−0.045	0.041	—					
6. Fp1θ	−0.304^***^	−0.333^***^	−0.129	−0.128	0.605^***^	—				
7. F7α2	0.278^**^	0.235^**^	0.197^*^	0.063	−0.506^***^	−0.252^***^	—			
8. T4α2	0.149	0.149	0.193^*^	0.116	−0.056	0.057	0.255^**^	—		
9. C4δ	−0.228^**^	−0.201^*^	0.035	0.05	0.726^***^	0.445^***^	−0.476^***^	0.168	—	
10. O1δ	−0.019	−0.106	−0.016	0.108	0.640^***^	0.276^**^	−0.52^***^	0.045	0.724^***^	—

*p < 0.05, **p < 0.01, ***p < 0.001.

#### 3.3.2 Potential associated pathways for muscle strength, resting EEG characteristics, working memory, and cognitive function

**Figure 2:**
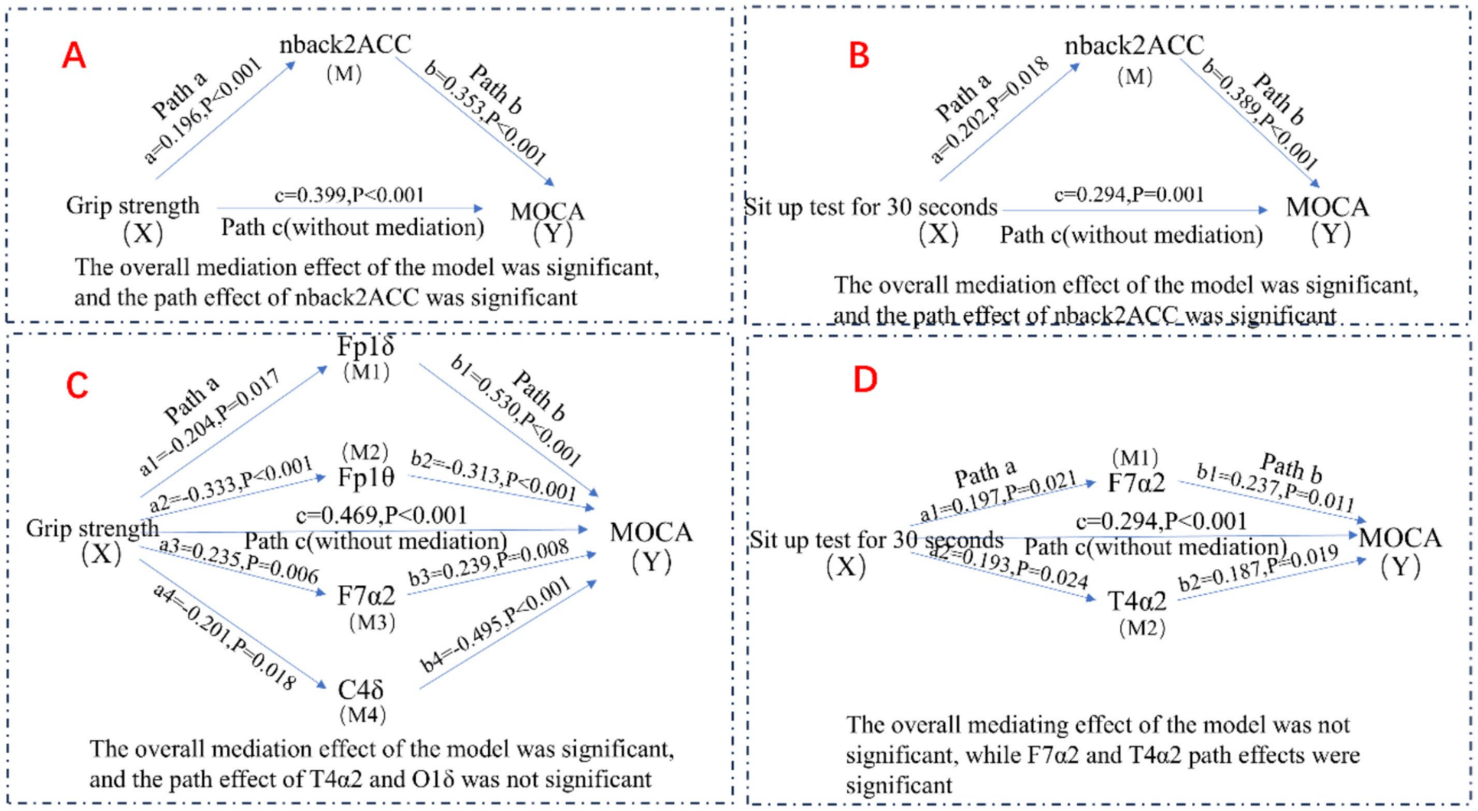
Potential association pathways for muscle strength, resting EEG characteristics, working memory, and cognitive function.

Building on the hierarchical regression analyses, we further explored potential pathways linking muscle strength, resting-state EEG features, working memory, and cognitive function. Mediation analyses were performed using Model 4 of the PROCESS macro, with covariates including age, BMI, and education controlled, to examine the mediating roles of resting-state EEG activity and working memory (see Figure 2).

As shown in Tables 5, 6, grip strength was significantly associated with cognitive performance, with a total effect of *β* = 0.469 (95% CI [0.469, 0.619]). The direct effect, controlling for the working memory mediator, remained significant (*β* = 0.399, 95% CI [0.399, 0.541], *p* < 0.001), while the indirect effect via working memory was also significant (*β* = 0.070, 95% CI [0.008, 0.136]), accounting for 14.93% of the total effect. Similarly, lower-limb strength (30 s chair stand test) showed a total effect of *β* = 0.294 (95% CI [0.294, 0.467]) on cognitive function. The direct effect remained significant (β = 0.216, 95% CI [0.216, 0.369]), and the indirect effect via working memory was β = 0.078 (95% CI [0.008, 0.167]), explaining 26.53% of the total effect.

**Table 5:** Regression analysis of the mediating model of working memory between muscle strength and cognitive function.

Outcome variable	Predictor variable	Model fit indices		Regression coefficients		
		R^2^	F	β	t	*P*
2-back task accuracy	Grip strength	0.038	5.425	0.196	2.329	<0.001
MOCA	Grip strength	0.219	38.034	0.468	6.167	<0.001
MOCA	Grip strength	0.340	34.531	0.399	5.579	<0.001
	2-back task accuracy			0.353	4.942	<0.001
2-back task accuracy	30s chair stand	0.041	5.717	0.202	2.391	0.018
MOCA	30s chair stand	0.086	12.815	0.294	3.580	0.001
MOCA	30s chair stand	0.232	20.197	0.216	2.795	<0.001
	2-back task accuracy			0.389	5.023	<0.001

**Table 6:** Bootstrap mediation effect tests for working memory.

Independent variable	Effect type	Effect	Bootstrap SE	Bootstrap 95%CI		*P*
				Lower	Upper	
Grip strength	Total effect	0.469	0.076	0.468	0.619	< 0.001
	Direct effect	0.399	0.072	0.399	0.541	< 0.001
	Mediated effect (working memory)	0.070	0.029	0.008	0.136	0.032
30s chair stand	Total effect	0.294	0.082	0.294	0.457	< 0.001
	Direct effect	0.216	0.077	0.216	0.369	< 0.001
	Mediated effect (working memory)	0.078	0.041	0.008	0.167	0.045

For the EEG-based model (Tables 7, 8), grip strength showed a total effect of *β* = 0.468 (95% CI [0.469, 0.619]), with a direct effect of β = 0.318 (95% CI [0.318, 0.465], *p* < 0.001), and a significant indirect effect via resting-state EEG features (β = 0.150, 95% CI [0.069, 0.261]), accounting for 32.05% of the total effect. Specific EEG markers—Fp1θ, F7α2, and C4δ—showed statistically significant mediation paths (see Table 8). In contrast, lower-limb strength also had a significant total effect on cognition (β = 0.294), with a direct effect of β = 0.213 (95% CI [0.213, 0.359], *p* < 0.001), but the indirect effect via EEG was non-significant (β = 0.081, 95% CI [−0.034, 0.200]), as the confidence interval included zero.

**Table 7:** Regression analysis of the mediating model of resting EEG characteristics between muscle strength and cognitive function.

Outcome variable	Predictor variable	Model FIT INDICES		Regression coefficients		
		R^2^	F	β	t	*P*
Fp1δ	Grip strength	0.042	5.880	−0.204	−2.425	0.017
Fp1θ	Grip strength	0.111	16.812	−0.333	−4.100	<0.001
F7α2	Grip strength	0.056	7.879	0.235	2.807	0.006
T4α2	Grip strength	0.022	3.053	0.149	1.747	0.083
C4δ	Grip strength	0.041	5.709	−0.201	−2.389	0.018
O1δ	Grip strength	0.011	1.532	−0.106	−1.238	0.218
MOCA	Grip strength	0.220	38.035	0.469	6.167	<0.001
MOCA	Grip strength	0.411	12.845	0.318	4.292	<0.001
	Fp1δ			0.530	4.341	<0.001
	Fp1θ			−0.313	−3.434	<0.001
	F7α2			0.239	2.718	0.008
	T4α2			0.160	2.081	0.039
	C4δ			−0.495	−4.104	<0.001
	O1δ			0.238	2.234	0.027
Fp1δ	30s chair stand	0.002	0.268	−0.045	−0.518	0.605
Fp1θ	30s chair stand	0.017	2.274	−0.129	−1.508	0.134
F7α2	30s chair stand	0.039	5.464	0.197	2.337	0.021
T4α2	30s chair stand	0.037	5.223	0.193	2.285	0.024
C4δ	30s chair stand	0.001	0.169	0.035	0.411	0.682
O1δ	30s chair stand	0.001	0.033	−0.016	−0.182	0.856
MOCA	30s chair stand	0.087	12.815	0.294	3.580	<0.001
MOCA	30s chair stand	0.368	10.706	0.213	2.887	0.005
	Fp1δ			0.564	4.479	<0.001
	Fp1θ			−0.381	−4.148	<0.001
	F7α2			0.237	2.574	0.011
	T4α2			0.187	2.367	0.019
	C4δ			−0.591	−4.749	<0.001
	O1δ			0.272	2.468	0.015

Using grip strength as an example, this table illustrates the mediation pathway through resting-state EEG features on cognitive performance (MoCA). Rows 1–6 represent the effects of grip strength on specific EEG channels (e.g., Fp1δ, Fp1θ, F7α2), corresponding to the first stage of the mediation process. Row 7 presents the direct effect of grip strength on MoCA after accounting for EEG features, while row 8 summarizes the total indirect effect via the combined EEG mediators. This decomposition highlights the contributions of individual brain regions and clarifies the neurophysiological mechanisms underlying the muscle strength–cognition association.

**Table 8:** Bootstrap mediation effect tests of resting EEG characteristics.

Independent variable	Effect type	Effect	Bootstrap SE	Bootstrap 95%CI		*P*
				Lower	Upper	
Grip strength	Total effect	0.468	0.076	0.469	0.619	< 0.001
	Direct effect	0.318	0.074	0.318	0.465	< 0.001
	Mediated effect (resting EEG)	0.150	0.049	0.069	0.261	0.002
	Fp1δ	−0.108	0.057	−0.236	−0.014	0.048
	Fp1θ	0.104	0.039	0.043	0.197	0.008
	F7α2	0.056	0.026	0.010	0.112	0.031
	T4α2	0.024	0.017	−0.005	0.062	0.158
	C4δ	0.110	0.057	0.006	0.225	0.048
	O1δ	−0.025	0.022	−0.070	0.019	0.256
30s chair stand	Total effect	0.294	0.082	0.294	0.467	< 0.001
	Direct effect	0.213	0.074	0.213	0.359	0.004
	Mediated effect (resting EEG)	0.081	0.058	−0.034	0.200	0.163
	Fp1δ	−0.025	0.049	−0.126	0.066	0.610
	Fp1θ	0.049	0.034	−0.013	0.122	0.150
	F7α2	0.047	0.025	0.007	0.103	0.040
	T4α2	0.036	0.021	0.004	0.083	0.048
	C4δ	−0.021	0.062	−0.151	0.101	0.735
	O1δ	−0.004	0.025	−0.045	0.060	0.873

In summary, these findings indicate that working memory significantly mediates the association between both upper and lower limb muscle strength and cognitive function, whereas resting-state EEG features mediate only the grip strength–cognition relationship. These results suggest that grip strength provides a unique neurophysiological pathway to cognition, while working memory serves as a broader cognitive mediator across different muscular systems.

## 4 Discussion

The study demonstrates significant associations between age, educational attainment, and cognitive impairment in older adults. Furthermore, multifactorial relationships involving resting-state EEG features; muscle strength; working memory; and cognitive functioning were identified, with their combination constituting key determinants of cognitive performance.

The study identified both advanced age and educational attainment as key determinants of cognitive decline. Aging emerged as a robust risk factor for cognitive deterioration in older adults, consistent with previous literature (Xin et al., 2025). Age-related changes—such as reductions in brain volume (particularly in the hippocampus and prefrontal cortex), diminished synaptic plasticity, neurotransmitter depletion, and cerebrovascular degeneration—have been well documented as contributors to cognitive vulnerability (Missonnier et al., 2004). These neurobiological alterations manifest as progressive deficits across multiple cognitive domains, including memory, processing speed, and executive function (He et al., 2023). In contrast, higher educational attainment exhibited protective effects, with greater years of education associated with reduced severity of cognitive impairment (Nie and Hu, 2025). Education may exert neuroprotective influences by enhancing neural network complexity and efficiency, thereby facilitating cognitive reserve mechanisms that buffer against neuropathological damage (Elkana and Beheshti, 2025). Epidemiological evidence indicates that each additional year of education is associated with a 7–11% reduction in dementia risk (Xu et al., 2016), with particularly pronounced benefits observed in verbal memory and executive functioning (Archer et al., 2018; Grande et al., 2016). Importantly, although age and education both significantly shape cognitive trajectories, they likely do so through distinct neurobiological mechanisms and interact differentially with other modifiers of cognitive aging. These findings underscore the need to consider both vulnerability and resilience factors in understanding individual variability in age-related cognitive outcomes.

This study demonstrated that resting-state EEG metrics—specifically Fp1δ, Fp1θ, F7α2, and C4δ in frontoparietal regions—along with muscular strength (upper and lower extremities) and working memory capacity, jointly predicted cognitive performance. These findings support our original hypothesis and provide novel electrophysiological evidence for the utility of EEG spectral features as biomarkers of cognitive impairment. The robust association between muscular strength, particularly handgrip, and cognitive function is consistent with prior studies (Xin et al., 2025; Cai et al., 2023; Nie and Hu, 2025; Sternäng et al., 2016; Burtscher and Burtscher, 2024). However, inconsistent results reported by Ritchie et al. (2016) may reflect methodological differences in cognitive assessment protocols and participant characteristics. Notably, oscillatory activity in resting-state frontal, parietal, and occipital cortices has been shown to couple with motor function (Ho et al., 2025), and specific EEG features—such as alpha-1 power at F7/F8 and T5/T6—are sensitive to interindividual differences in muscular strength (Xin et al., 2025). Working memory also emerged as a key cognitive predictor, with its decline reflecting neurodegenerative changes in the structural and functional integrity of prefrontal, temporal, and parietal regions. These findings reinforce the multifactorial etiology of cognitive impairment, wherein neuromuscular decline and cognitive dysfunction may be linked via shared pathophysiological pathways, including low-grade inflammation, insulin resistance, oxidative stress, and dysregulation of neurotrophic factors (Dercon et al., 2021; Gregorio and Battaglia, 2024; Tanaka et al., 2024; Nazzi et al., 2024; Zabetian-Targhi et al., 2021).

This study found that both resting-state EEG features (in the frontoparietal regions) and working memory independently mediated the relationship between muscle strength and cognitive decline; however, no significant joint mediation effect was observed between EEG and working memory. In the EEG mediation model, frontoparietal oscillatory activity (Fp1δ, Fp1θ, F7α2, C4δ) emerged as a key neurophysiological factor underlying the muscle–cognition association. Previous research suggests that muscle strength may enhance cognitive function by upregulating activation in specific prefrontal regions, and that older adults with higher levels of physical activity and greater muscle strength exhibit a 30–46% reduced risk of cognitive decline (Herold et al., 2019; Lubitz et al., 2017). In the working memory model, working memory significantly mediated the association between muscle strength and cognitive performance. As a core dimension of cognition (Elliott et al., 2011; Bopp and Verhaeghen, 2020), the capacity to retain information in working memory is essential for maintaining cognitive resilience in older age (Seo and Lee, 2022). Notably, no significant correlation was found between working memory and resting-state EEG features. We speculate that this discrepancy may stem from excessive cognitive load during the working memory task. The participants in this study had a mean age of 72.65 ± 7.754 years and exhibited substantial cognitive impairment, which may have rendered the 2-back task too demanding for their cognitive capacity, potentially introducing measurement bias. Therefore, future studies should incorporate larger samples and varied working memory load levels to validate and expand upon these findings.

Our findings revealed that handgrip strength exerted a more pronounced effect on resting-state EEG features and working memory performance, while lower-limb muscle strength did not significantly mediate EEG-cognition associations. Specifically, grip strength demonstrated robust positive correlations with working memory performance (reaction speed and accuracy) (Mavros et al., 2017), as well as synergistic associations with higher-order cognitive domains such as verbal fluency and episodic memory (Nie and Hu, 2025; Ruan et al., 2020). Its decline may serve as an early warning marker for working memory deterioration (Liu et al., 2022). This phenomenon likely stems from the unique physiological specialization of the upper-limb neuromuscular system (Gabriel et al., 2006; Zhang et al., 2023), where the hand acts as a primary effector of fine motor control and somatosensory feedback. Its function is tightly coordinated by prefrontal and parietal cortical regions. Grip training may therefore enhance cognitive performance through mechanisms such as improved synaptic plasticity and increased secretion of brain-derived neurotrophic factor (BDNF), facilitating more efficient engagement of cognition-related neural networks (Veronese et al., 2016; Chou et al., 2019).

In contrast, lower-limb muscle strength exhibited a weaker mediating role in EEG activity. This may be attributable to distinct corticospinal control pathways, reduced relevance of lower-limb precision in standard cognitive assessments, and the relatively limited cognitive demands imposed by gross motor tasks (Xin et al., 2025; Duchowny et al., 2022). Additionally, the lack of significance may partially reflect insufficient statistical power due to modest sample size. Future studies should expand participant cohorts to further elucidate the neurophysiological underpinnings linking lower-limb strength, brain oscillatory dynamics, and cognitive function.

## 5 Limitations and future prospects

Several limitations merit acknowledgment. First, the cross-sectional design precludes causal inference; thus, mediation pathways should be interpreted as associative rather than mechanistic. Second, although adjustments were made for key covariates (e.g., age, BMI, education), residual confounding from unmeasured factors—such as diet quality, sleep architecture, vascular integrity, and habitual physical activity—cannot be excluded. Prospective studies incorporating ecological momentary assessment and multimodal biomarkers (e.g., actigraphy, omega-3 index) are warranted. Third, while ICA and visual inspection attenuated major EEG artifacts, formal regression of physiological signals (e.g., ECG, EMG) was not performed, limiting signal specificity. Fourth, absolute spectral power was favored over ratio metrics (e.g., theta/alpha) to enhance robustness in resting-state elderly EEG, though the latter may offer complementary insights and should be considered in future analyses. Finally, functional status was not directly assessed via standardized ADL instruments (e.g., Barthel Index, Lawton IADL), limiting our ability to contextualize neuromuscular-cognitive interplay in daily living domains.

## 6 Conclusion

Upper-limb muscle strength predicts cognitive function by enhancing prefrontal-parietal network efficiency and optimizing working memory, whereas lower-limb strength shows limited association. These findings suggest that grip strength may serve as a modifiable biomarker and potential target for interventions aimed at preserving cognitive health in aging populations. However, causal relationships cannot be inferred due to the cross-sectional design. Future longitudinal studies are warranted to validate these mediation pathways and assess the efficacy of targeted neuromuscular interventions.

## Data Availability

The original contributions presented in the study are included in the article/Supplementary material, further inquiries can be directed to the corresponding author.
